# Unveiling the Potential of Cannabinoids in Multiple Sclerosis and the Dawn of Nano-Cannabinoid Medicine

**DOI:** 10.3390/pharmaceutics16020241

**Published:** 2024-02-07

**Authors:** Roua A. Nouh, Ahmed Kamal, Oluwaseyi Oyewole, Walaa A. Abbas, Bishoy Abib, Abdelrouf Omar, Somaia T. Mansour, Anwar Abdelnaser

**Affiliations:** 1 Biotechnology Graduate Program, School of Sciences and Engineering, The American University in Cairo, P.O. Box 74, New Cairo 11835, Egypt; roua@aucegypt.edu (R.A.N.); oyewole488@aucegypt.edu (O.O.); wadly@aucegypt.edu (W.A.A.); abdelroufomar@aucegypt.edu (A.O.); 2 Biochemistry Department, Faculty of Science, Suez University, P.O. Box 43221, Suez 43533, Egypt; ahmed.kamal@sci.suezuni.edu.eg; 3 Department of Chemistry, School of Sciences and Engineering, The American University in Cairo, P.O. Box 74, New Cairo 11835, Egypt; bishoyabib@aucegypt.edu (B.A.); somaia_tawfik@aucegypt.edu (S.T.M.); 4 Institute of Global Health and Human Ecology, School of Sciences and Engineering, The American University in Cairo, P.O. Box 74, New Cairo 11835, Egypt

**Keywords:** multiple sclerosis, autoimmune disease, cannabinoids, tetrahydrocannabinol, cannabis, treatment modalities, immunomodulatory, nanomedicine

## Abstract

Multiple sclerosis is the predominant autoimmune disorder affecting the central nervous system in adolescents and adults. Specific treatments are categorized as disease-modifying, whereas others are symptomatic treatments to alleviate painful symptoms. Currently, no singular conventional therapy is universally effective for all patients across all stages of the illness. Nevertheless, cannabinoids exhibit significant promise in their capacity for neuroprotection, anti-inflammation, and immunosuppression. This review will examine the traditional treatment for multiple sclerosis, the increasing interest in using cannabis as a treatment method, its role in protecting the nervous system and regulating the immune system, commercially available therapeutic cannabinoids, and the emerging use of cannabis in nanomedicine. In conclusion, cannabinoids exhibit potential as a disease-modifying treatment rather than merely symptomatic relief. However, further research is necessary to unveil their role and establish the safety and advancements in nano-cannabinoid medicine, offering the potential for reduced toxicity and fewer adverse effects, thereby maximizing the benefits of cannabinoids.

## 1. Introduction

Multiple sclerosis (MS) is an autoimmune disorder that affects the central nervous system (CNS) [1] and is one of the leading causes of neurological impairment in teenagers and adults [2]. Multiple sclerosis can be mainly categorized into three types: Relapsing-Remitting MS (RRMS), Secondary Progressive MS (SPMS), and Primary Progressive MS (PPMS). Most MS patients (85–90%) initially present with RRMS, with around 90% eventually transitioning to SPMS and the remaining 10% experiencing PPMS [3]. Multiple sclerosis (MS) is distinguished by the presence of muscle spasms, spasticity, neuropathic pain, bladder dysfunction, tremors, dysarthria, and cognitive impairments, such as memory disturbances [4]. There is currently a growing trend in utilizing cannabis for therapeutic purposes as a symptomatic treatment. Numerous trials and patients have reported that it may be beneficial in managing and controlling symptoms associated with multiple sclerosis (MS). This review will examine the conventional treatment of multiple sclerosis (MS), the increasing interest in using cannabis as a treatment method for MS, its suggested mode of action, commercially available therapeutic cannabinoids, and the innovative use of cannabis in nanomedicine.

## 2. Treatment Modalities for MS Management

Multiple sclerosis is characterized by CNS plaques, demyelination, gliosis, and inflammation [5]. The CNS plaques consist of inflammatory cells, including lymphocytes, demyelinated axons, decreased oligodendrocytes, severed axons, and increased astrocyte proliferation resulting in gliosis [6], which is found primarily in white matter, as well as in gray matter [7]. Gliosis can be considered a prominent feature of MS plaques and is regarded as a secondary response to CNS damage, which can endure for weeks or months following brain injury, resulting in the fibrous proliferation of glial cells in the affected CNS areas and the formation of fibrous scars [8,9,10]. The medications utilized for managing multiple sclerosis are categorized into two primary groups: Treatments that modify the course of the disease and treatments that alleviate symptoms [11]. Due to MS pathogenesis, disease-modifying therapies change the course of the disease by controlling or regulating the immune system. These treatments have an anti-inflammatory effect, mainly during the relapse phase of MS, reducing the frequency of relapses, slowing the buildup of lesions in MRI scans, and, in some cases, modestly enhancing the improvement of disability [12]. However, symptomatic treatments aim to decrease the symptoms, but they are limited by their toxicity [13]. For instance, the long-term use of analgesics for pain management in MS raises concerns due to its potential hepatotoxic effects and the risk of harmful overdose, particularly in cases of severe pain [14]. This exemplifies the risk of relying on symptomatic treatments for chronic conditions, underscoring the vital role of disease-modifying therapies. It also emphasizes addressing the core issue rather than merely managing resultant symptoms.

Disease-modifying therapeutics (DMTs) have been extensively researched to control MS progression and improve MS patients’ quality of life. Betaseron, a preparation of interferon beta-1b, was the first treatment for RRMS to be approved by the FDA in 1993 [15]. Over the next two decades, there was a significant shift in the approach to treating multiple sclerosis, as numerous novel therapies were introduced to the pharmaceutical market [16]. As of August 2023, the National MS Society reported significant progress in multiple sclerosis (MS) treatment, with the FDA approving 24 disease-modifying therapies. Notably, the approved treatments encompass five distinct interferon beta (IFN-β) drugs—Avonex, Betaseron, Extavia, Plegridy, and Rebif—two formulations of glatiramer acetate (Copaxone and Glatopa) and six monoclonal antibodies—Kesimpta, Briumvi, Lemtrada, Ocrevus, Tysabri, and Tyruko. More than ten other drugs, including fingolimod, dimethyl fumarate, teriflunomide, and the chemotherapeutic agent mitoxantrone, have also been approved [17].

**Figure 1:**
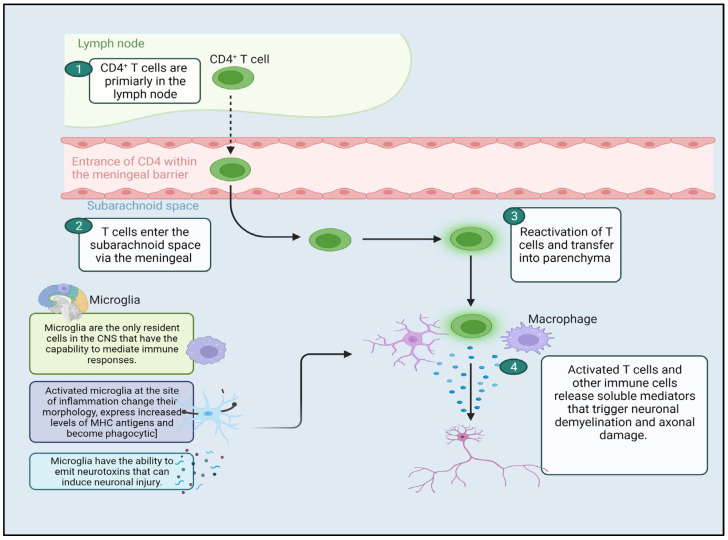
Schematic illustration of CD4^+^ T-cells and their involvement in the pathophysiology of Multiple sclerosis. CD4: Cluster of differentiation 4, Microglia: Immune cells of central Nervous system, created with Biorender.

The pathogenesis of MS is believed to progress through three phases: first, the generation of autoreactive T-cells directed against myelin occurs. Next, these autoreactive T-cells cross the blood-brain barrier (BBB) regulated by endothelial cells. Lastly, these T-cells attack the central nervous system, leading to oligodendrocyte demyelination. These phases are depicted in Figure 1 [18]. Beta-interferons, specifically interferon-β1a (IFN-β1a) and interferon-β1b (IFN-β1b), are types of type I interferons that have received FDA approval for managing MS. They function by modulating the immune system [19]. This is achieved by reducing Major Histocompatibility Complex (MHC) molecule expression on Antigen-Presenting Cells (APCs) and shifting from pro-inflammatory to anti-inflammatory cytokines. IFN-b also suppresses T-cell growth and prevents the movement of inflammatory cells into the central nervous system [20].

Several IFN beta-based drugs have been developed, such as Betaseron (IFN-β1b), Avonex (IFN-β1a), and Extavia (IFN-β1b) [21]. Copaxone, a Glatiramer acetate (GA) formulation, on the other hand, works in a different mechanism by combining four amino acids in its structure (L-alanine, L-glutamic acid, L-lysine, and L-tyrosine) in a random sequence, forming a mixture closely resembling the antigenic properties of myelin basic protein, which is a crucial part of the myelin sheath that covers nerve fibers [22]. The FDA approved GA for use in MS in 1996 [23]. Its prevailing belief is that it promotes the activation of myelin-targeting T-lymphocyte suppressor cells. It is also believed to counteract detrimental T-cell activity by preventing specific immune cells from presenting antigens. Many believe that GA’s capacity to encourage a change in the immune environment from a pro-inflammatory Th1 response to an anti-inflammatory one is at the heart of its mechanism of action [24,25]. Table 1 shows the most recognized and utilized mechanisms targeted by DMTs, along with some of the mainly prescribed disease-modifying treatments for multiple sclerosis.

## 3. Cannabinoids and the Endocannabinoid System (ECS)

### 3.1. Cannabinoids

*Cannabis sativa*, *Cannabis indica*, and *Cannabis ruderalis* are the three most common species of the cannabis plant, which is in the Cannabaceae family [32]. The cannabis plant has a long history of practical uses, including as a food and oil source and even in producing paper and linen, two of man’s necessities [33]. In addition, its psychoactive qualities enabled its use in medical surgeries, even though its components and mechanism of action in the human body were unknown at the time [34]. Phytocannabinoids, endocannabinoids, and synthetic cannabinoids are the three primary sources of more than sixty cannabinoids with physiological effects [8]. The cannabis plant contains more than 100 phytocannabinoids, including the two most significant ones, which are Δ^9^-tetrahydrocannabinol (THC) and cannabidiol (CBD). It is believed that Δ^9^-THC is the primary psychoactive compound found in cannabis [9].

### 3.2. The Endocannabinoid System (ECS)

The primary impact of cannabinoids occurs through the endocannabinoid system (ECS), which consists of a set of signaling pathways regulated by cannabinoid receptors cannabinoid-1 (CB1) and cannabinoid-2 (CB2). The activation of these pathways is commonly triggered by the attachment of endogenous cannabinoids (endocannabinoids) like Anandamide (AEA) and 2-Arachidonoyl Glycerol (2-AG) to the CB1 and CB2 receptors [35]. The CB1 receptors are situated primarily in nerve terminals and function to inhibit the release of neurotransmitters. Conversely, CB2 receptors are predominantly located in immune cells. Their role encompasses regulating cytokine production and migrating immune cells within and beyond the central nervous system [36,37]. The endocannabinoid system (ECS) is essential for maintaining the body’s homeostasis by regulating the balance between the inhibitory and excitatory states of the nerves. This is accomplished by activating CB1 receptors located on inhibitory GABAergic and excitatory glutamatergic presynaptic terminals, inhibiting neurotransmitter release [38]. Additionally, the ECS is responsible for many physiological and pathological processes in the body. It controls biological mechanisms, such as pain, food intake, anxiety, and memory [39].

## 4. Neuroprotection Effect of Cannabinoids

**Figure 2:**
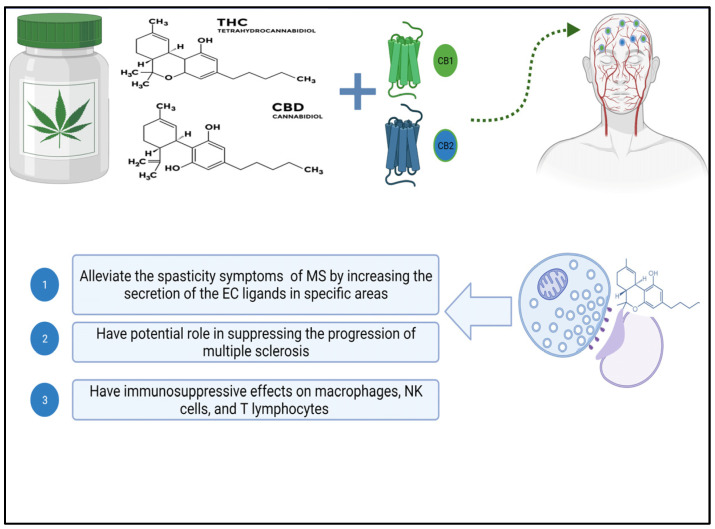
Diagram depicting the biological effects of cannabis’ active ingredients on multiple sclerosis. Abbreviations for cannabinoid receptors 1 and 2, tetrahydrocannabinol, and cannabidiol created with Biorender.

The neuroprotective effect of cannabinoids in multiple sclerosis (MS) may be attributed to their role in regulating the excessive excitability of neurons in the central nervous system (CNS). The CB1 receptor is located predominantly in GABAergic neurons within the hippocampus. It is also found in neurons that use glutamate as a neurotransmitter and in astrocytes and subcellular compartments [40,41]. The release of cholinergic and dopaminergic neurotransmitters is regulated by cannabinoid signaling and the regulation of excitatory/inhibitory transmission by CB1 receptors, as shown in Figure 2 [42,43]. Studies have shown that cannabinoid-based therapy can effectively reduce symptoms of multiple sclerosis, such as spasticity, pain, gallbladder dysfunction, and tremors [44], which is achieved by increasing the secretion of endocannabinoids in targeted areas, activating CB1 receptors, and limiting the release of neurotransmitters from presynaptic terminals, which results in a reduction in the excessive excitatory state in the neurons and a potential neuroprotection effect of the CNS [45]. In addition, cannabinoids’ impact on the regulation and modulation of microglial cells within the CNS has been investigated. Inflammation has been shown to elevate CB2 receptors in glial and immune cells, even though they are less prevalent in the healthy brain, as observed in EAE models [46].

Moreover, blood samples from MS patients exhibited higher levels of pro-inflammatory cytokines and excessive expression of CB1 and CB2 receptors [47]. These findings, along with the observation that activating CB2 receptors reduces the secretion of TNF-α and oxidative free radicals within the brain, underscore the critical role of the ECS signaling pathway and cannabinoids in controlling CNS inflammation through immuno-regulatory functions in neurons. Furthermore, this plays a vital role in the neuroprotection of the CNS by mitigating oxidative stress. Interestingly, the part of cannabinoids in neuroprotection could also be due to their antioxidant effect. Preliminary factors in neurodegenerative diseases include oxidative stress, which occurs when reactive oxygen or nitrogen species surpass antioxidants. CBD, being a phenolic compound, exhibits reactive oxygen-scavenging properties. In experimental studies on PC12 cells, CBD demonstrated approximately 50% higher antioxidant activity than vitamins. It effectively reduced oxidative stress caused by reactive oxygen species (ROS) by limiting lipid peroxidation and inhibiting the accumulation of ROS products. Additionally, CBD reduced induced cell-apoptosis factors, such as DNA fragmentation and caspase-3 activation. These findings highlight the potential neuroprotective effects of CBD against oxidative damage in neurodegenerative conditions [48].

## 5. Immunomodulatory Effect of Cannabinoids

The presence of cannabinoid receptor 2 (CB2) in white blood cells has sparked interest in the ability of cannabinoids to regulate the immune system. THC binds to CB1 receptors in the brain, whereas CB2 receptors are found predominantly in immune cells in the peripheral nervous system. The precise role of the endocannabinoid system in immune regulation is not yet fully comprehended, despite evidence of cannabinoids affecting immune cell function [49,50]. According to a study by Nichols et al. in 2020, cannabidiol (CBD) has been recognized as an anti-inflammatory substance and has some characteristics of suppressing the immune system [51]. Exposure to high concentrations of cannabis can impair immune responses, according to in vitro and in vivo research. This reduces the activity and cytokine production capacity of macrophages, natural killer cells, and T lymphocytes [52]. However, rather than reducing immune system activity, an adequate amount of cannabis in the body increases lymphocyte metabolic activity and boosts the production of pro-inflammatory cytokines [53]. These dose-dependent cannabinoid activities point to the biphasic effect of cannabis constituents [52]. Despite this potential biphasic effect of cannabinoids, CBD has been shown in several studies to act as an immunomodulator during inflammation, regulating the inflammatory response by influencing various inflammatory cascades involving both anti-inflammatory and pro-inflammatory mediators, as discussed in the study by Furgiuele et al. [54]. Inflammation, axonal demyelination, and symptoms like spasticity and pain are all helped by these neuroprotective mechanisms. Using EAE murine models of multiple sclerosis, researchers found that CBD, with the help of myeloid-derived suppressor cells (MDSCs), improved EAE progression dose-dependently. According to the research conducted by Elliott et al., CBD had several effects, including a decrease in T-cell proliferation in the central nervous system (CNS) and a decrease in the pro-inflammatory cytokines IL-17 and IFNγ [55]. Additionally, CBD treatment decreased inflammation and axonal loss in multiple sclerosis models engineered with myelin oligodendrocyte glycoprotein (MOG) to imitate EAE. The reason for this was that CBD inhibits the infiltration of T-cells and the activation of microglial cells, as reported in the study by Kozela et al. [56].

## 6. Therapeutic Potential of Cannabinoids

Cannabidiol has demonstrated encouraging effects in treating a range of medical conditions. Within the domain of epilepsy therapy, CBD has shown efficacy as an anticonvulsant medication, particularly in the treatment of severe childhood epilepsy syndromes such as Dravet syndrome and Lennox–Gastaut syndrome. Furthermore, CBD has been studied for its potential as an antidepressant, antipsychotic, and anxiolytic agent. Moreover, CBD has demonstrated an anticancer effect. While further research is necessary to comprehend its advantages fully, CBD exhibits promise as a reliable and efficient medication, indicating therapeutic implications for inflammation, neuroprotection, epilepsy, depression, and pain [57,58]. These findings support the potential therapeutic benefits of cannabinoids in managing neuroinflammation and its impact on MS-related pathology. Research has investigated the potential of cannabinoids to inhibit the progression of multiple sclerosis (MS) and provide neuroprotection in animal models. The results have varied under different experimental conditions, as specified in Table 2, and there have yet to be any human trials conducted with appropriate doses. CBD has demonstrated effectiveness in animal MS models and human cells tested in a laboratory setting. However, its impact on the immune system of MS patients is yet to be observed [59]. Individual variance and genetic polymorphism may point to distinct processes or responses to cannabinoids, leading to a reasoned explanation.

## 7. Commercial Therapeutic Cannabinoids in Use

A variety of cannabis-based medications have received approval for the treatment of various diseases, such as multiple sclerosis, as indicated in Table 3. The initial cohort of human cannabinoids to be granted regulatory approval by the medical community consists of the synthetic THC called “Dronabinol” and the THC analog known as “Nabilone” [68,69]. Nabilone exhibited greater efficacy compared to THC due to its ability to induce a more substantial reduction in cAMP levels within the brains of rodents [70]. The US Food and Drug Administration (FDA) approved Dronabinol and Nabilone in the 1980s, specifically for the treatment of chemotherapy-induced nausea and vomiting, as well as anorexia in AIDS patients, resulting in weight loss [71]. Furthermore, multiple ongoing studies aim to understand better the practicality of using Dronabinol or Nabilone for pain management. These studies include evaluating the effectiveness of dronabinol for treating neuropathic back pain and assessing the potential of Nabilone for managing acute pain in individuals with inflammatory bowel disease [72].

Notably, Nabilone demonstrated efficacy in addressing sleep-related symptoms of post-traumatic stress disorder (PTSD) in a limited pilot study involving military personnel [73]. Dronabinol also decreased anorexia, disturbed behavior [74], and nighttime agitation [75] in Alzheimer’s disease. Clinical trials are evaluating Dronabinol [76] and Nabilone [77] in treating agitation associated with Alzheimer’s disease. Research into the use of synthetic THC and THC-like compounds for various health issues has yielded encouraging outcomes for the benefit of cannabinoids for the management of multiple sclerosis.

There is substantial evidence and multiple clinical studies to substantiate the use of these medications for patients with multiple sclerosis (MS), as well as their approved applications for managing chronic pain, cannabis use disorder (CUD), and weight loss in individuals with AIDS [78,79,80,81]. The advancement of a compound that resembles THC shows significant potential. An effective strategy that has demonstrated potential is the utilization of a blend of THC and CBD, specifically in the context of alleviating pain and spasticity. The synergy of THC and CBD is advantageous due to their distinct mechanisms of action. THC selectively binds to CB1/2 receptors in the spinal, supraspinal, and peripheral pain systems, enhancing its effectiveness [82]. The combined effects of THC and CBD can be observed in Sativex, an oromucosal spray comprising 25 mg of CBD and 27 mg of 9-THC in a 1.0 mL aromatized water-ethanol mixture. Several clinical trials have been carried out to evaluate the efficacy of Sativex as an adjunctive treatment for controlling moderate to severe spasticity in patients diagnosed with multiple sclerosis [81,83]. It has been demonstrated to decrease spasticity, improving the patient’s quality of life. Recently, spasticity related to multiple sclerosis and cancer pain has been recognized as an approved use for plant-derived THC in the form of an oromucosal spray known as Nabiximols or Sativex [84]. Neuropathic pains, a common symptom of MS that affects between 17% and 70% of patients, were also shown in a study to be reduced by Sativex.

Additionally, Sativex is well tolerated by MS patients and has a low incidence of side effects [85]. Regarding the impact of CBD alone, a recent preclinical study has discovered that a 7-day treatment with CBD normalized the disrupted 5-HT neurotransmission, reduced mechanical allodynia, and diminished anxiety-like behavior in a model of neuropathic pain [86]. Another recent study has also shed light on the impact of CBD on experimental autoimmune encephalomyelitis (EAE), a murine model of multiple sclerosis. The study found that CBD reduced neuroinflammation by lowering pro-inflammatory cytokines, boosting anti-inflammatory cytokines, and elevating the levels of myeloid-derived suppressor cells [55].

**Table 3:** Commercial cannabinoids for therapeutic use.

Drug Name	FDA Approval	Active Constituents	Indication	Ref.
Epidiolex	2018	Purified CBD formulation.	Treatment of seizures associated with Lennox–Gastaut syndrome (LGS), Dravet syndrome, or tuberous sclerosis complex (TSC).	[87]
Nabiximols	No	Combination of CBD (cannabidiol) and THC (delta-9-tetrahydrocannabinol).	Management of spasticity associated with MS.	[88]
Ajulemic Acid	No	Synthetic THC-11-oic acid analogue.	Management of chronic neuropathic pain by selectively binding to CB2 receptor.	[89]
Sativex	No	1:1 ratio of Δ-9-tetrahydrocannabinol (THC) and cannabidiol.	Management of spasticity in MS patients.	[90]
Cesamet	1985	Derivative of Δ-9-tetrahydrocannabinol.	Treatment of chemotherapy-induced nausea with cancer patients.	[91]
Marinol	1985	Synthetic THC analogue	Management of chemotherapy-induced nausea and vomiting. Treatment of anorexia associated with immune deficiency patients.	[92]
Nabiximols	No	Combination of CBD and THC.	Pain management of cancer and MS patients.	[93]
Dronabinol	1985	Synthetic delta-9-tetrahydrocannabinol.	Management of neuropathic pain.	[94]
Nabilone	1985	Synthetic THC	Management of Parkinson’s disease and chemotherapy-induced nausea and vomiting.	[95]

## 8. Nanomedicine and Cannabinoids for MS Treatment

Nanotechnology in drug delivery (nanomedicine) offers the advantage of directing the active components of the medication to specific target areas, resulting in sustained release and improved treatment outcomes [96]. Nanomedicine has shown the potential to overcome the limitations of conventional medicines. Utilizing nanotechnology allows for targeted release and precise control over the dosage, leading to improved treatment efficacy and decreased toxicity. Nanomaterials in therapeutic systems have become a promising approach for treating MS, offering both neuroprotection and increased effectiveness by crossing the blood-brain barrier (BBB) [97].

### 8.1. Enhancing Drug Stability and Solubility through Nanomedicine

Nanomedicine greatly influences the chemical properties of loaded drugs, particularly in terms of their stability and solubility. Nanosuspensions and nanotechnology have been demonstrated to augment the solubility and stability of drugs in drug delivery systems, consequently enhancing their bioavailability and bioactivity. Nanoparticles can alter the pharmacokinetics of drugs, leading to improved drug safety and efficacy. Additionally, nanomedicine can address issues such as poor aqueous solubility, poor permeation, low systemic availability, and instability of drugs [98,99,100,101,102]. For instance, the administration of nanosuspension drugs with low water solubility is an advancing and swiftly expanding domain, garnering heightened interest for its potential to mitigate toxicity and enhance drug effectiveness by eliminating the need for co-solvents in the formulation [103,104].

Moreover, notable progress has been made in nanomedicine, particularly in improving nanoformulations’ stability using different stabilizers. A comprehensive review by Wu et al. underscored drug nanoparticles’ physical and chemical stability, elucidating the mechanisms and corresponding characterization techniques crucial for sustaining their stability. This advancement significantly enhances the advantages of employing nanotechnology in drug delivery [102]. This reformulation of pre-existing medicines or the development of new ones has been substantially boosted by the increasing research in nanomedicine, leading to changes in drug toxicity, solubility, and bioavailability profiles [100,101]. In conclusion, nanomedicine is pivotal in bolstering the stability and solubility of encapsulated drugs, ultimately enhancing their overall efficacy and safety.

### 8.2. Mechanism of Nano-Cannabinoids Evading the Blood-Brain Barrier

The blood-brain barrier (BBB) comprises a specialized network of endothelial cells, pericytes, and astrocytes, acting as a defense mechanism to prevent the extravasation of materials. Tight junctions between adjacent brain endothelial cells limit paracellular transport, restricting the passive entry of molecules to a narrow range of size and lipophilicity. These formidable physical and functional barriers impede the exposure of drugs to intracranial tissues. Tight junctions commonly exclude hydrophilic small molecules from entering the brain, and although many lipophilic drugs can passively diffuse, their penetration into diseased brain tissue is often inefficient. This inefficiency typically necessitates high drug doses, leading to dose-limiting systemic toxicity [105,106]. In light of the numerous challenges associated with the passage of small molecules through the blood-brain barrier, nanoparticles have been investigated as a potential means to enhance drug delivery to brain tissues [107]. Much of the current research has concentrated on improving the passive transport mechanisms of drug-loaded nanoparticles across the BBB. For example, in diseases where the BBB is compromised, such as glioblastoma, nanostructures have been observed to extravasate through leaky vasculature, accumulating at tumor sites.

Similarly, strategies have been developed to enhance drug delivery across an intact BBB by initially disrupting this barrier [108,109,110,111]. However, such approaches, while allowing unregulated passage across the BBB, may compromise the BBB’s homeostatic functions and expose the brain to harmful toxins and pathogens [112]. In contrast, alternative approaches for diseases like SHH subgroup medulloblastoma, where the BBB remains intact, involve the use of nontargeting nanocarriers to prolong the systemic circulation of small-molecule drugs, and over a relatively long time, an appreciable number of drugs would cross the BBB. Nevertheless, this has only partially improved on-target toxicity profiles at high doses [112]. Notably, recent research suggests that the passive entry of nanoparticles into solid tumors through gaps between endothelial cells is a minor mechanism, with up to 97% of transport occurring through an active process across endothelial cells [113]. A recent study by Tylawsky et al. focused on exploring transendothelial transport facilitated by caveolin-1-dependent transcytosis using nanoparticles that specifically target P-selectin receptors on endothelial cells. This transport mode occurs through brain endothelial cells that maintain an intact blood-brain barrier (BBB). Given these findings, an alternative approach involves leveraging a precise receptor–ligand interaction to enable targeted and controlled delivery of therapeutic agents encapsulated within nanoparticles across an intact BBB [114]. Regarding cannabinoids, high levels of P-glycoprotein (P-gp) may reduce the brain delivery of cannabinoids, but decreasing P-gp activity could cause cerebral accumulation. These findings suggest that the use of nanoparticles targeting specific proteins to enhance transcytosis while modulating P-gp activity could potentially facilitate the delivery of nano-cannabinoids across the blood-brain barrier, offering a promising avenue for the treatment of central nervous system disorders [114,115].

### 8.3. Nano-Cannabinoids: Challenges and Potentials

**Figure 3:**
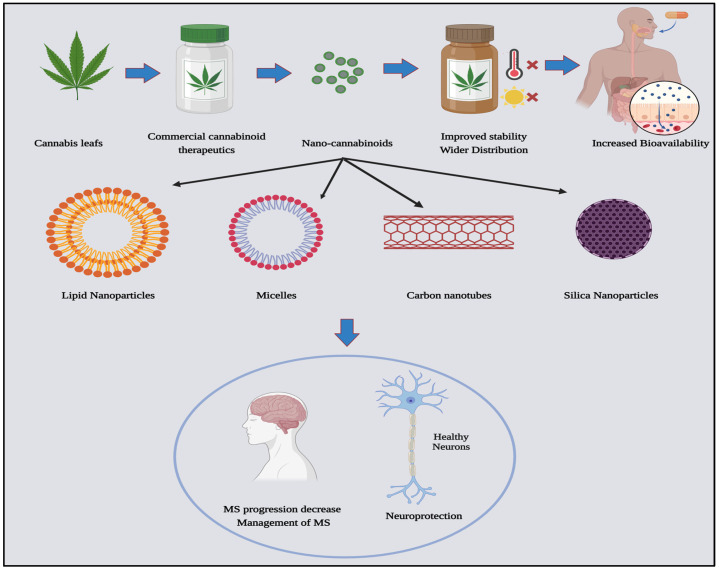
Nano-cannabinoids: types and potentials in medicine, created with Biorender.

Currently, there is no single conventional treatment that works for all MS patients at all stages, and the limited use of FDA-approved cannabinoids in medical practice is due to the inconsistent efficacy, inadequate targeting, and the absence of a thorough understanding of the stability of commercially available cannabis formulations [96,116,117]. Cannabinoids, being lipophilic, have varying oral absorption and distribution levels [118]. Additionally, fluctuations in temperature and exposure to light can result in the rapid degradation of cannabinoids [119]. The limited bioavailability of cannabinoids is attributed to low absorption rates of 20 to 30% for oral administration and 10–to 60% when inhaled, increasing the need for higher dosages, which may result in toxicity, especially for normal non-targeted tissues. Cannabinoids are also prone to auto-oxidation and degradation, influenced by factors such as light or temperature. All these factors hinder the widespread use of cannabinoid formulations in MS [120,121]. However, scientists have attempted to overcome the limitations of traditional cannabinoid treatments by incorporating them into nano-based therapeutic systems. This integration aims to improve the stability of cannabinoids, reduce the required dosage for MS treatment, and evade the BBB, thereby targeting MS lesions with minimal toxicity and fewer side effects for normal neural tissues and cells [122]. Several studies have formulated various nano-cannabinoid types, including lipid nanoparticles, micelles, silica nanoparticles, and carbon nanotubes [123]. This is depicted in Figure 3.

A study by Aparicio-Blanco et al. found that cannabinoid-based nanomaterials and formulations have demonstrated their efficacy against glioma cells. The researchers effectively integrated CBD, a significant compound found in cannabis, into Solid Lipid Nanocapsules (LNCs) through two methods, firstly, by incorporating CBD into the core of the LNCs, and secondly, by decorating the surface of the LNCs with CBD. Both techniques resulted in a slower, sustained release of CBD, decreased the required IC50 for treatment, and effectively targeted glioma cells [124]. Moreover, Δ9-tetrahydrocannabinol (Δ9-THC) is recognized for its antitumor activity. Recently, Duran-Lobato et al. conducted a noteworthy study in which they created and evaluated nanoparticles encapsulated with Δ9-THC and targeted to cannabinoid receptors [125]. Surprisingly, their technique demonstrated more potential for controlling anticancer effects and the sustained release of cannabinoids at target cells. Recently, multiple studies have shown that encapsulating lipophilic compounds like cannabinoids in nanostructured delivery systems can effectively cross the blood-brain barrier (BBB) with improved targeting ability to the CNS [125]. This increases the bioavailability, solubility, and stability of the drug in the body over a more extended period [126,127]. While cannabinoid-based nanomedicine holds numerous advantages, it has not yet achieved widespread acceptance as a fully developed treatment for patients with multiple sclerosis. There is a crucial need for additional research to further explore and understand its potential benefits and effectiveness, particularly in the context of multiple sclerosis.

## 9. Concluding Remarks and Perspectives

Multiple sclerosis (MS) is an autoimmune disease of the central nervous system (CNS) [1] and is one of the leading causes of neurological impairment in teenagers and adults [2]. Currently, there is no single conventional treatment that works for all MS patients at all stages of the disease, but some treatments aim to slow down the progression and relieve painful symptoms [117]. The potential role of cannabinoids in suppressing MS progression and neuroprotection has been studied in animal models and human cell lines. Studies have shown that cannabinoid-based therapy can effectively reduce symptoms of multiple sclerosis, such as spasticity, pain, gallbladder dysfunction, and tremors, and therefore, they have been considered a symptomatic treatment [44]. Interestingly, cannabinoids have also shown anti-inflammatory effects, immunosuppressive properties, and neuroprotection capability in MS [51]. Several clinical trials have studied the use of cannabinoids to manage MS [78,79,80,81]. The molecular effect of cannabinoids in MS and their function in neuroprotection and immunomodulation suggest that cannabinoids are more than a symptomatic treatment. They can be considered disease-modifying treatment (DMT) if given the optimal dose. Cannabinoids, being lipophilic, have varying oral absorption and distribution levels [118].

Additionally, fluctuations in temperature and exposure to light can result in the rapid degradation of cannabinoids [119]. Therefore, several studies have investigated the potentiality of nanoparticles encapsulated with cannabinoids. The promising results have shown increasing bioavailability, solubility, and sustainable release of the drug in the body over a more extended period [126,127]. Consequently, cannabinoid-based nanotechnology could provide a more targeted approach to treating MS lesions with less toxicity and decreased adverse effects. However, further research is needed to establish the safety and advancements in nano-cannabinoid medicine.

## Figures and Tables

**Table 1:** The most prescribed disease-modifying treatments (DMTs) for multiple sclerosis.

Drug Generic Name	FDA Approval	Chemical Composition	Mechanism of Action	Refs.
Betaseron Extavia	1993, 1995 2009	Interferon-β1b (IFN-β1b)	Modulate the immune system. Reduce MHC molecule expression on APCs. Shift from pro-inflammatory to anti-inflammatory cytokines. Suppress T-cell growth. Prevent inflammatory cell movement into the CNS.	[17,21]
Avonex Rebif Plegridy	1996 1998 2014	Interferon-β1a (IFN-β1a)		
Copaxone Glatopa	1996 2015	Glatiramer acetate (GA)	Mimic myelin basic protein. Stimulate T-lymphocyte suppressor cells targeting myelin antigen. Disrupt antigen-presenting capability of immune cells, countering harmful T-cell activity. Shift immune environment from pro-inflammatory Th1 response to anti-inflammatory response.	[17,23,24,25]
Kesimpta Briumvi Lemtrada Ocrevus Tysabri Tyruko	2020 2022 2014 2017 2004 2023	Ofatumumab (Anti-CD20) Ublituximab (Anti-CD20) Alemtuzumab (Anti-CD52) Ocrelizumab (Anti-CD20) Natalizumab (Anti-α4β1 integrin) Natalizumab (Anti-α4β1 integrin)	Deplete B cells, hindering their movement to the CNS and reducing antigen presentation to T-cells. Modulate B-cell secretion of pro-inflammatory cytokines. Reduce B-cell activation and differentiation into immunoglobulin-secreting plasmablasts.	[17,26,27,28,29,30]
Novantrone	2000	Mitoxantrone	Interacts with DNA, causing single- and double-stranded breaks, and inhibits DNA repair via topoisomerase II suppression. Affects proliferating cells like B and T lymphocytes. Reduces secretion of IFN-γ, TNF-α, and IL-2. Induces apoptosis in B lymphocytes and monocytes.	[19,31]
Gilenya Tascenso ODT Mayzent Zeposia	2010 2021 2019 2020	Fingolimod Fingolimod Siponimod Ozanimod	S1P receptors modulators Retain lymphocytes in lymphoid ogans and prevents infiltration into the CNS.	[12,17]

**Table 2:** Clinical studies on the use of cannabinoids for the management of multiple sclerosis.

Treatment Used	Experimental Design	Results	Ref.
A daily dose of CBD (75 mg/kg) for 5 days	In vivo (mice)	Dimension in the T-cell infiltration and neuroinflammation in the brain and spinal cord’s white matter pathways.	[60]
A daily dose of CBD (10 mg/kg/day i.p.)	In vivo (mice)	Decrease the proliferation of T-cells.	[61]
A daily dose of CBD + THC (10 mg/kg/day i.p.)	In vivo (mice)	(CBD + 9-THC) Decrease the number of CD3^+^ T-cells, CD3^+^ CD4^+^ T-cells, and demyelination. Furthermore, the combination of THC and CBD improves the clinical symptoms of MS patients.	[61]
A daily dose of CBD (20 mg/kg/day i.p.)	In vivo (mice)	Clinical symptoms have a delayed onset and are less severe.	[55]
Determination of T-cells in marijuana smokers	In vivo (human subjects)	Reduction in the T-cells proliferation.	[50]
A dose of 10^−5^ to 10^−4^ of delta 8-THC + delta 9- THC + CBD	In vitro (Animal cell culture)	Decrease the proliferation of T-cells.	[62]
Three intraperitoneal (i.p.) injections of CBD (5 mg/kg, one per day) given at the outset of clinical disease	In vitro (cell culture, T-cell line)	Reduction in disease symptoms during the days following the injections, as well as a significant delay in disease development. Additionally, prevention of T-cell proliferation.	[56]
A dose (10/100 ng/mL) of Δ-THC	In vitro (B cells)	Increase the proliferation of T-cells.	[63]
After MS induction, CBD (10 mg/kg/day i.p.) was given for 7 days	In vitro (CD4^+^ T lymphocytes)	CD4^+^ T-cells’ pro-inflammatory phenotype is reversed.	[64]
A dose of CBD (0.1–1.5 μM)	In vitro (T-cell line derived from lymph node cells)	CD4^+^ T-cells and CD19^+^ B cells succumbed more frequently, whereas CD11b^+^ monocytes did not.	[65]
CBD (5–10 mg/kg/3 times per week or 50 mg/kg/day i.p.)	In vivo (mice)	Clinical symptoms and tissue lesions are reduced dose-dependently.	[66]
A dose (5–10 μg/mL) of THC	In vitro (cell culture)	Decrease in the number of Natural killer cell (NK).	[50]
Dose of 8 μg/mL or 2.6 × 10^−5^ M of THC	In vitro (animal cell culture)	Inhibition of T-cell proliferation.	[67]
10^−5^ to 10^−4^ M concentrations of delta8, and delta9-tetrahydrocannabinol (THC)	In vitro (human cell culture)	Reduction in the proliferation of T-cells.	[62]

## Data Availability

All figures (Figure 1, Figure 2 and Figure 3) in this manuscript were constructed using Biorender (www.biorender.com). Additionally, a publication license was obtained from Biorender for using these figures in this manuscript, which allows for the reproduction and distribution of the figures.
